# Hypothalamic Neuroendocrine Circuitry is Programmed by Maternal Obesity: Interaction with Postnatal Nutritional Environment

**DOI:** 10.1371/journal.pone.0006259

**Published:** 2009-07-16

**Authors:** Hui Chen, David Simar, Margaret J. Morris

**Affiliations:** 1 Department of Pharmacology, School of Medical Sciences, University of New South Wales, New South Wales, Australia; 2 Department of Medical and Molecular Bioscience, Faculty of Science, University of Technology, Sydney, Australia; 3 Health and Exercise Science, School of Medical Sciences, University of New South Wales, New South Wales, Australia; The Research Center of Neurobiology - Neurophysiology of Marseille, France

## Abstract

**Objective:**

Early life nutrition is critical for the development of hypothalamic neurons involved in energy homeostasis. We previously showed that intrauterine and early postnatal overnutrition programmed hypothalamic neurons expressing the appetite stimulator neuropeptide Y (NPY) and suppressor proopiomelanocortin (POMC) in offspring at weaning. However, the long-term effects of such programming and its interactions with post-weaning high-fat-diet (HFD) consumption are unclear.

**Research Design and Methods:**

Female Sprague Dawley rats were exposed to chow or HFD for 5 weeks before mating, throughout gestation and lactation. On postnatal day 1, litters were adjusted to 3/litter to induce postnatal overnutrition (vs. 12 in control). At postnatal day 20, half of the rats from each maternal group were weaned onto chow or HFD for 15 weeks. Hypothalamic appetite regulators, and fuel (glucose and lipid) metabolic markers were measured.

**Results:**

Offspring from obese dams gained more weight than those from lean dams independent of post-weaning diet. Maternal obesity interacted with post-weaning HFD consumption to cause greater levels of hyperphagia, adiposity, hyperlipidemia, and glucose intolerance in offspring. This was linked to increased hypothalamic NPY signaling and leptin resistance in adult offspring. Litter size reduction had a detrimental impact on insulin and adiponectin, while hypothalamic NPY and POMC mRNA expression were suppressed in the face of normal energy intake and weight gain.

**Conclusions:**

Maternal obesity, postnatal litter size reduction and post-weaning HFD consumption caused obesity via different neuroendocrine mechanims. There were strong additive effects of maternal obesity and post-weaning HFD consumption to increase the metabolic disorders in offspring.

## Introduction

Both genetic and environmental factors can contribute to obesity and gene-diet interactions are also important [1]. The “thrifty-genotype” promotes fat deposition in times of plenty to enable survival in times of famine [2]. This appears to represent a disadvantage under the current conditions of food abundance, as overconsumption of energy-rich food makes significant contributions to global obesity. Animal studies suggest that the influence of overconsumption of high-fat diet (HFD) is so powerful that it can override genetic factors to promote obesity and metabolic disorders, such as insulin resistance [3]. This may partially explain the worldwide rise in obesity [4].

The hypothalamus plays a key role in energy homeostasis. The most commonly studied hypothalamic appetite regulators are the robust appetite stimulator neuropeptide Y (NPY) and appetite suppressor proopiomelanocortin (POMC), produced predominantly in the arcuate nucleus (Arc). Hypothalamic NPY concentrations are elevated before a meal to stimulate appetite, and continuous or repeated central administration of NPY leads readily to obesity, while POMC derived α-melanocyte-stimulating hormone (MSH) counteracts NPY to inhibit feeding and promote negative energy balance [5]. The hypothalamic NPY and POMC expressing neurons are plastic, being modified by chronic overconsumption of HFD, as shown by us and others, and are associated with the development of metabolic disorders, such as increased adiposity and hyperinsulinaemia [6], [7], [8], [9].

Increasing evidence suggests that both maternal phenotype and nutritional state in the early postnatal phase are important in promoting obesity in offspring. Offspring from obesity-prone rats remained obese even when they were suckled by lean dams [3]. They developed adiposity and impaired glucose and lipid metabolism as early as postnatal day 20 [10], [11], [12], which were maintained until adulthood [13]. Overnutrition during the lactation period can also cause an obese phenotype, even in obesity-resistant rats. When these rats were suckled by obese-prone dams with rich milk, they developed obesity on post-weaning HFD [3]. Increased milk availability through litter size reduction also leads to obesity [12], [14]. Thus maternal obesity and postnatal overfeeding can promote offspring obesity. We showed that the impact of maternal obesity appears to be amplified by early postnatal overfeeding, as pups from obese dams showed exaggerated adiposity and glucose intolerance at weaning if they were raised in small litters [12]. This was closely linked to an alteration in hypothalamic NPY and POMC expression, as well as their functional receptors [12].

In the rat, differentiation of the neuronal systems regulating energy homeostasis begins during gestation, and continues until weaning [15]. Nutritional changes over this period modify the expression of appetite regulators and in offspring from obesity-prone rats, the hypothalamic neuron projections were shown to be permanently disrupted [16]. HFD feeding during pregnancy increased the proliferation of hypothalamic orexigenic peptide-producing neurons [17]. Previously, we showed that established maternal obesity and litter size reduction reciprocally altered hypothalamic NPY and POMC mRNA expression [12]. These findings suggest that modifications to the hypothalamic homeostasis circuitry may be a fundamental mechanism underlying the increased risk of obesity by overnutrition during critical windows of early development. Supporting this hypothesis, a maternal ‘junk food’ diet in pregnancy and lactation promoted an exacerbated preference for ‘junk food’ in offspring [11], [13].

However, it remains unclear how post-weaning diet (chow or HFD) can interact with programmed alterations in hypothalamic metabolic circuitry following early life overnutrition. We hypothesized that maternal obesity and litter size reduction would cause different metabolic responses to post-weaning dietary interventions (standard rodent chow or HFD), through distinct adaptations of hypothalamic and peripheral markers that regulate energy homeostasis. To examine this question, hypothalamic appetite regulators, adiposity, parameters of glucose and lipid metabolism were measured in 18 week old male offspring.

## Methods

The study was approved by the Animal Ethics Committee of the University of New South Wales.

### 1. Maternal obesity

Female outbred Sprague Dawley rats aged 9 weeks (Animal Resource Centre WA, Australia) were housed at 20±2°C and maintained on a 12:12 h light/dark cycle. Two groups of rats paired for body weight were exposed to either standard laboratory chow (control group, 11 kJ/g, energy 14% fat, 21% protein, 65% carbohydrate, Gordon's Specialty Stockfeeds, NSW, Australia), or palatable cafeteria-style HFD (HFD group, 15.3 kJ/g, energy 34% fat, 18% protein, and 50% carbohydrate), as described previously [12]. After 5 weeks females were mated. After confirmation of pregnancy, they were housed individually and monitored throughout gestation. Dams consumed the same diet until pups reached 20 days (weaning).

### 2. Postnatal litter size adjustment and post-weaning HFD feeding

**Figure 1:**
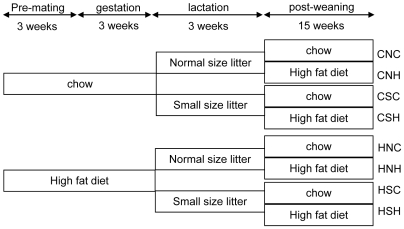
Experimental groups. Rats were fed chow or high fat diet prior to congestion and during gestation and lactation. The offspring were raised in either normal (12) or small (3) size litters. At weaning, rats were fed either chow or high fat diet. The first letter describes maternal diet, second letter litter size and the third letter postweaning diet. CNC: chow-fed mother-normal size litter-postweaning chow diet. CNH: chow-fed mother-normal size litter-postweaning high-fat diet. CSC: chow-fed mother-small size litter-postweaning chow diet. CSH: chow-fed mother-small size litter-postweaning high-fat diet. HNC: high-fat diet-fed mother-normal size litter-postweaning chow diet. HNH: high-fat diet-fed mother-normal size litter-postweaning high-fat diet. HSC: high-fat diet-fed mother-small size litter-postweaning chow diet. HSH: high-fat diet-fed mother-small size litter-postweaning high-fat diet.

On day 1 after birth, litters were adjusted to 3 pups (small litter, to increase milk availability) versus normal litter size maintained at 12 pups (sex ratio 2∶1) as described previously (Fig. 1) [14]. This yielded four groups: chow-fed dam with normal-size litter (CN) and small-size litter (CS), HFD-fed dam with normal-size litter (HN) and small-size litter (HS). Animals that littered on the same day were distributed between dams from the same diet group, thus using cross-fostering, pups littered by 8–9 mothers within the same diet group were distributed across litter groups. In order to yield enough male offspring, 7–8 dams were used to suckle CS and HS (with 3 pups per dam), while CN and HN offspring were suckled by 3 dams. Body weight of pups was monitored every 3 days. At 20 days, half the male pups from each litter were weaned onto chow, while the other half were given palatable HFD. This yielded 8 groups: CNC, CNH, CSC, CSH, HNC, HNH, HSC, HSH (Fig. 1). Body weight and energy intake was measured weekly. All the adult offspring in each group were used to measure the body weight, energy intake, intraperitoneal glucose tolerance test (IPGTT), organ parameters at death, and plasma hormones. Rats from different litters within each group were used to measure mRNA expression.

### 3. IPGTT [14]

Sixteen week old animals were weighed and fasted for 5 hours. After the establishment of a baseline glucose level (Accu-Chek^®^ glucose meter; Roche Diagnostics, Nutley, USA) rats were administered 2 g glucose/kg (i.p.). Blood samples were taken at 15, 30, 60, and 90 minutes to monitor glucose. The area under the curve (AUC) was calculated for each animal.

### 4. Sample collection

At 18 weeks, after overnight (14 h) fasting, rats were deeply anesthetized (ketamine/xylazine 180/32 mg/kg, i.p.). After measurement of naso-anal (N-A) length, blood was collected by cardiac puncture and blood glucose was measured immediately. Plasma was stored for hormone (leptin, insulin, and adiponectin) and triglyceride (TG) measurements. Then animals were killed by decapitation and the hypothalamus was microdissected on ice into areas containing the arcuate nucleus (Arc), paraventricular nucleus (PVN), and posterior hypothalamus (PH). The anterior hypothalamus was removed following coronal sections at the optic chiasm and 1.5 mm caudal. This slice was hemisected into a dorsal segment (PVN), and a ventral segment (Arc) which was combined with a triangular ventral portion of a more caudal section (Bregma −2.3 mm to −3.8 mm). The dorsal region of this section comprised PH. Brain samples were snap frozen in liquid nitrogen, and stored at −80°C for determination of mRNA expression of genes of interest. Body fat (brown adipose tissue (BAT), epididymal fat, retroperitoneal (Rp) fat, mesenteric fat was dissected and weighed, as well as various organs (heart, liver, and pancreas). Rp fat and skeletal muscles were kept to provide peripheral metabolic markers. Tibia length was measured as a marker of growth.

### 5. Plasma TG, leptin, insulin, and adiponectin assays

Plasma TG was measured using glycerol standard (equivalent to 0–8.46 mmol•l^−1^ TG, Sigma, St. Louis, MO, USA) and TG reagent (Roche Diagnostics, Nutley, NJ, USA). Samples and standards were incubated with TG reagent at 37°C for 20 min, and read on a microplate reader (BIO-RAD 680XR, Hercules, CA, USA) at 490 nm. Plasma leptin, insulin and adiponectin concentrations were measured using commercial radioimmunoassay kits (Linco, St. Charles, Missouri, USA). The insulin resistance index was estimated by HOMA: fasting plasma insulin (ng/ml) × fasting plasma glucose (mM)/(22.5×0.0417) (the greater the HOMA value, the greater the level of insulin resistance [18], [19]), as well as leptin∶adiponectin ratio [20], [21].

### 6. Quantitative real-time PCR

Total RNA was isolated from the hypothalamus, Rp fat, and skeletal muscle using TriZol reagent (Invitrogen Australia Pty Limited, Melbourne, VIC, Australia). The purified total RNA was used as a template to generate first-strand cDNA using M-MLV Reverse Transcriptase, RNase H-, Point Mutant Kit (Promega, Madison, WI, USA). TaqMan probe/primers (Applied Biosystems, Foster City, CA, USA) that were designed and validated by the manufacturer were used for quantitative real-time PCR (Eppendorf Realplex 2, Eppendorf AG, Hamburg, Germany) (Table 1). Appetite regulator (NPY, POMC, agouti-related protein (AgRP), Y1 receptor, melanocortin-4 receptor (MC4R), single minded gene (Sim)1, corticotropin-releasing hormone (CRH), and markers of leptin signaling (Ob-Rb, signal transduction involves activation of transcription (STAT)3, suppressor of cytokine signaling (SOCS)3) were measured in the relevant hypothalamic sections. Markers of lipid oxidation (carnitine palmitoyl-transferase (CPT)-1, adipose triglyceride lipase (ATGL)) were measured in Rp fat; uncoupling protein 3 (UCP3) and adiponectin receptor (AdipoR)1 were measured in muscle. The probes for target genes were labeled with FAM and those for the housekeeping gene 18s were labeled with VIC. Gene expression was quantified in a single multiplexing reaction, where target genes were standardized to 18s rRNA. An individual sample from the control group was arbitrarily assigned as a calibrator against which all other samples were expressed as fold difference.

**Table 1:** TaqMan probe sequence (Applied Biosystem, Foster City, USA) used for real time-PCR.

Gene	NCBI gene references	FAM-labeled Probes (5′→3′)	Applied Biosystem Assay
AdipoR1	NM_207587.1,BC061838.1,DQ148391.1	CATATGGTTCCAGTCTCATCAGATT	Rn01114954_g1
ATGL	XM_341960.3	GCCTGCCTGGGCGAAGCGGGTGCCA	Rn01479969_m1
CPT-1	NM_031559.1,L07736.1,U88294.1,BC072522.1	CCAGGAGAGTGCCAGGAGGTCATAG	Rn00580702_m1
CRH	NM_031019.1,X03036.1	CAAGGGAGGAGAAGAGAGCGCCCCT	Rn01462137_m1
Ob-Rb	NM_012596.1	TTAATTTCCAAAAGCCTGAAACATT	Rn01433205_m1
MC4R	NM_013099.2	AGCAGAAGCCTGATTCCACTGTTTA	Rn01491866_s1
NPY	NM_012614.1	GCCCGCCCGCCATGATGCTAGGTAA	Rn00561681_m1
POMC	NM_139326.2	AAGCAACCTGCTGGCTTGCATCCGG	Rn00595020_m1
SOCS3	NM_053565.1	ACCCCCGGAGCACGCAGCCAGTGCC	Rn00585674_s1
STAT3	NM_012747.2	ACCCAGGTAGTGCTGCCCCTTACCT	Rn00562562_m1
UCP3	NM_013167.2,AB006614.1,AF030163.1, AF035943.1,U92069.1,BC072546.1	CAGGGGACTGTGGAAAGGGACTTGG	Rn00565874_m1
Y1 receptor	Z11504.1	TCATATGCTACTTCAAGATATACGT	Rn01402912_g1

### 7. Statistical methods

Results are expressed as mean±S.E.M. Data on body weight of pups over time was analyzed using analysis of variance (ANOVA) with repeated measures, followed by post hoc Fisher's Least Significance Difference (LSD) tests. Fat and organ weights, blood and plasma hormone concentrations, mRNA expression in all tissues were analyzed using factorial ANOVA, if the data were normally distributed. If not, data were log transformed to achieve normality of distribution before they were analyzed using factorial ANOVA.

## Results

### Effect of HFD feeding on breeders

HFD-fed female breeders consumed more than twice the energy of chow-fed breeders, resulting in 23% greater body weight before mating. At weaning, HFD-fed dams remained heavier than chow-fed mothers, with increased Rp, ovarian and mesenteric fat mass (Table 2) both as net weight and when data were standardized by body weight. Although blood glucose levels were similar across groups, plasma leptin and insulin concentrations were more than doubled in HFD-fed breeders (Table 2).

**Table 2:** Effect of HFD feeding on body parameters in breeders at kill.

	chow fed breeder	HFD fed breeder
	n = 11	n = 9
BW (g)	360.8±10.6	474.3±34.1 *
Liver (g)	12.32±0.39	13.00±1.07 *
Rp fat (g)	4.03±1.00	14.29±1.26 *
Ovarian fat (g)	5.86±1.04	17.68±4.19 *
Mesenteric fat (g)	7.88±1.35	20.72±2.21*
Blood glucose (mmol·l^−1^)	11.45±0.77	12.35±1.21
Plasma insulin (ng·ml^−1^)	0.11±0.04	0.30±0.09 *
Plasma leptin (ng·ml^−1^)	8.55±1.37	17.34±1.00 *

Results are expressed as mean±S.E.M. Data were analysed by *Student's* unpaired *t* test.

* Significantly different from chow fed breeders, p<0.05.

BW: body weight; HFD: high fat diet.

### Weight gain in offspring during development

**Figure 2:**
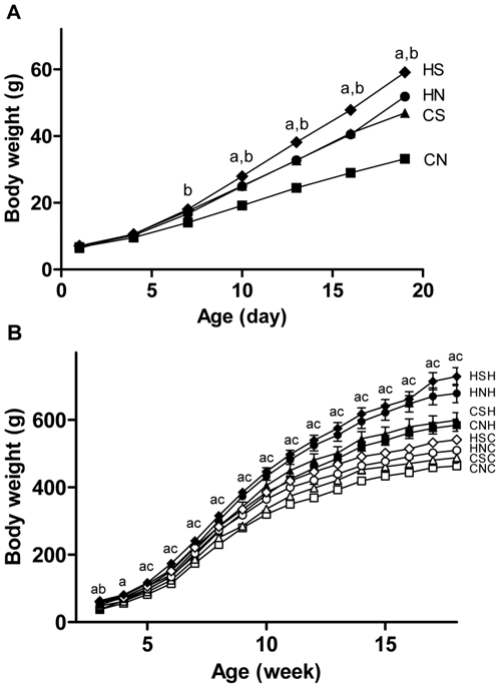
Body weight changes during the suckling and post-weaning periods. (A) the suckling period, (B) post-weaning period. Results are expressed as mean±S.E.M. Data were analysed by ANOVA with repeated measures, followed. P<0.05, a: maternal diet effect, compared with the offspring from chow-fed mothers with the same size litters; b: litter effect, compared with the offspring from mothers fed the same kind of diet with normal size litters; c: post-weaning diet effect, compared with chow-fed litter mates. (A). CN: square, n = 24; CS: triangle, n = 24; HN: circle, n = 22; HS: diamond, n = 20. (B). CNC: open square n = 12: CNH solid square n = 12; CSC: open triangle n = 12; CSH: solid triangle, n = 12; HNC: open circle, n = 11; HNH: solid circle, n = 11; HSC: open diamond, n = 10; HSH: solid diamond, n = 10. CNC: chow-fed mother-normal size litter-postweaning chow diet. CNH: chow-fed mother-normal size litter-postweaning high-fat diet. CSC: chow-fed mother-small size litter-postweaning chow diet. CSH: chow-fed mother-small size litter-postweaning high-fat diet. HNC: high-fat diet-fed mother-normal size litter-postweaning chow diet. HNH: high-fat diet-fed mother-normal size litter-postweaning high-fat diet. HSC: high-fat diet-fed mother-small size litter-postweaning chow diet. HSH: high-fat diet-fed mother-small size litter-postweaning high-fat diet.

During the suckling period, pups from obese dams became significantly heavier than those from lean dams from postnatal day 10 in both normal and small litters (P<0.05, Fig. 2). From postnatal day 7, pups raised in small litters grew faster than those in normal litters within the same maternal group (Fig. 2A). The body weight of CS pups overlapped that of HN pups until postnatal day 16, after which the latter became significantly heavier. Litter size reduction and maternal obesity interacted with each other to cause markedly increased body weight (HS group, Fig. 2A) during suckling.

Within only 2 weeks of weaning, HFD caused significant increases in body weight in all groups compared with their littermates receiving standard low fat chow (P<0.05, CNH *vs.* CNC, CSH *vs.* CSC, HNH *vs.* HNC, HSH *vs.* HSC, Fig. 2B). Energy intake in HFD-fed rats was approximately doubled compared to chow (Table 3), consistent with our previous findings [6], [7].

**Table 3:** Effects of maternal diet, postnatal litter size adjustment and post-weaning diet on body weight, length, organ mass, and adiposity at 18 weeks.

Postweaning diet Maternal diet & litter size	chow				HFD			
	CN	CS	HN	HS	CN	CS	HN	HS
	n = 12	n = 12	n = 11	n = 10	n = 12	n = 12	n = 11	n = 10
Body weight (g)	460.3±8.8	482.7±15.7	505.6±8.5*	524.8±6.6*	573.3±20.3‡	603.7±23.5‡	690.6±26.3* ‡	706.6±24.8* ‡
EI (kJ·24 h^−1^·rat^−1^)	283.9±21.2	294.2±22.2	308.9±23.4*	327.6±23.8*	594.7±50.6‡	577.5±46.6‡	680.7±58.2* ‡	674.3±54.1* ‡
N-A length (cm)	25.6±0.2	26.0±0.2	26. 5±0.2	26.5±0.2	26.8±0.3‡	27.1±0.4‡	27.2±0.2‡	27.4±0.2‡
Tibia (cm)	4.63±0.05	4.67±0.03	4.71±0.04*	4.71±0.02*	4.78±0.04‡	4.76±0.05‡	4.87±0.05* ‡	4.88±0.05* ‡
Liver (g)	11.5±0.4	12.1±0.4	12.3±0.3*	13.6±0.3*	16.5±0.8‡	17.0±1.1‡	19.8±1.3* ‡	20.7±1.0 * ‡
Heart (g)	1.16±0.04	1.36±0.03	1.26±0.06*	1.49±0.05*	1.24±0.02‡	1.55±0.05‡	1.36±0.03* ‡	1.64±0.06* ‡
BAT (g)	0.34±0.03	0.37±0.02	0.35±0.02*	0.43±0.03*	0.76±0.04‡	0.72±0.05‡	1.00±0.10* ‡	1.10±0.10* ‡
Rp fat (g)	3.05±0.24	4.32±0.44	4.30±0.27*	5.50±0.48*	11.79±1.20‡	12.34±1.41‡	20.94±2.18* ‡	19.77±1.26* ‡
Epididymal (g)	5.15±0.24	7.15±0.51	7.25±0.33*	8.20±0.38*	16.01±1.50‡	19.06±1.74‡	27.58±2.50* ‡	26.30±1.59* ‡
Mesenteric (g)	3.59±0.22	4.91±0.35	4.78±0.32*	5.30±0.32*	9.88±1.20‡	10.08±1.45‡	16.20±1.49* ‡	17.80±1.33* ‡

Results are expressed as mean±SEM. Data were analysed by multi-factor ANOVA.

P<0.05.

* maternal diet effect, compared with the offspring from chow-fed mothers with the same size litters.

‡ post-weaning diet effect, compared with chow-fed litter mates.

CN: chow-fed mother with normal size litter.

CS: chow-fed mother with small size litter.

HN: high-fat diet-fed mother with normal size litter.

HS: high-fat diet-fed mother with small size litter.

Maternal obesity caused a significant 9–14% increase in 24 h energy intake compared with that of offspring from lean dams raised in the same size litters and fed the same diet (Table 3). There was also a significant interaction between maternal obesity and postnatal HFD in increasing energy intake (P<0.05). The weight gain of offspring from obese dams appeared more pronounced than those from lean dams (Fig. 2B). After weaning, rats from small and normal litters had similar daily energy intakes of chow or HFD (Table 3). There was no litter size effect on body weight from postnatal week 4 (Fig. 2B).

### Glucose homeostasis in offspring

**Figure 3:**
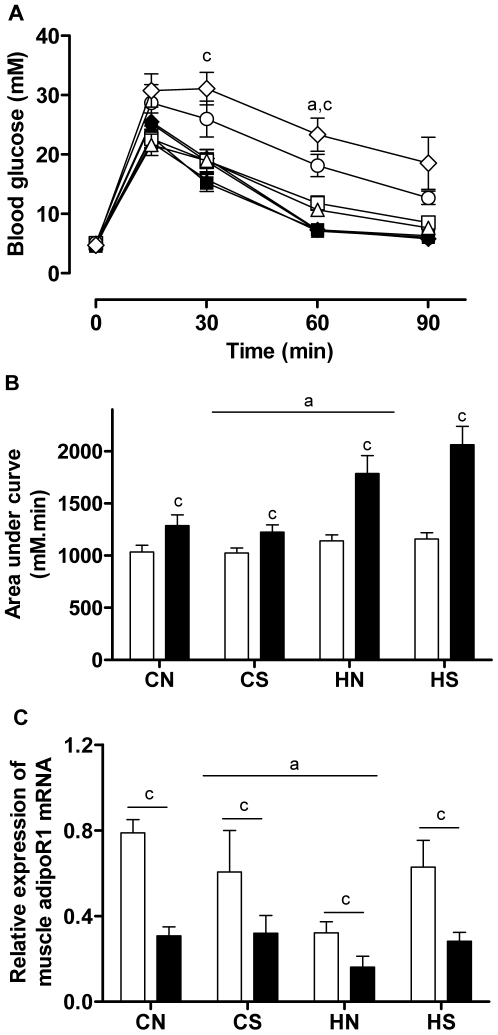
Glucose tolerance test and muscle AdipoR1 mRNA expression. (A) IP glucose tolerance test, (B) area under the curve at 16 weeks (glucose 2 g/kg, n = 10–12 per group), and (C) muscle AdipoR 1 mRNA expression (6–8 per group). Results are expressed as mean±S.E.M. Data in (A) were analysed by ANOVA with repeated measures followed by a post hoc LSD test. Data in (B) (C) were analysed by multi-factor ANOVA. P<0.05, a: maternal diet effect, compared with the offspring from chow-fed mothers with the same size litters; c: post-weaning diet effect, compared with chow-fed litter mates. (A). CNC: open square n = 12: CNH solid square n = 12; CSC: open triangle n = 12; CSH: solid triangle, n = 12; HNC: open circle, n = 11; HNH: solid circle, n = 11; HSC: open diamond, n = 10; HSH: solid diamond, n = 10. Maternal groups are shown on X axis in (B) and (C). Post-weaning chow diet: open bars; post-weaning HFD: closed bars. CN: chow-fed mother with normal size litter. CS: chow-fed mother with small size litter. HN: high-fat diet-fed mother with normal size litter. HS: high-fat diet-fed mother with small size litter.

Fasting blood glucose was at similar higher levels in all HFD-fed groups (P<0.001, HFD effect). However, HFD consumption caused significant glucose intolerance in offspring from both lean and obese dams (Fig. 3A,B); this was more pronounced in those from obese dams, reflected by higher blood glucose levels at 15 min and 60 min during IPGTT and 50% greater AUC, indicating delayed uptake. Offspring from obese dams displayed glucose intolerance and increased AUC even when they were fed chow, compared with those from lean dams (Fig. 3A,B). Rats raised in small size litters showed similar IPGTT responses as those raised in normal size litters within the same maternal group (Fig. 3A,B).

HFD consumption resulted in 2–3 times higher plasma insulin concentrations in offspring from lean dams, and interacted with maternal obesity to cause nearly a 5 times increase in these offspring (P<0.001, HFD effect and interaction with maternal obesity, Table 4); a significant effect of litter size was also observed in plasma insulin in offspring consuming HFD (P<0.05, litter effects, Table 4). On the other hand, although a maternal effect on plasma adiponectin levels was only evident in offspring consuming a HFD, adiponectin was lower after being standardized by fat mass (P<0.05, Table 4). Post-weaning HFD interacted with maternal obesity to increase the HOMA index and leptin:adiponectin ratio, and reduce adiponectin/g Rp fat levels (P<0.05, HFD effect and interaction with maternal obesity). When all groups were combined, the leptin:adiponectin ratio was positively correlated with AUC, HOMA, and plasma TG (r = 0.56, 0.81, 0.67 respectively, all P<0.0001, n = 80). Muscle AdipoR1 expression was downregulated by HFD consumption and maternal obesity (P<0.05, HFD and maternal effect, Fig. 3C).

**Table 4:** Effects of maternal diet, postnatal litter size adjustment and post-weaning diet on plasma TG, glucose, and hormones.

Postweaning diet Maternal diet & litter size	Chow				HFD			
	CN	CS	HN	HS	CN	CS	HN	HS
	(n = 12)	(n = 12)	(n = 11)	(n = 10)	(n = 12)	(n = 12)	(n = 11)	(n = 10)
TG (mmol·l^−1^)	0.35±0.05	0.53±0.07	0.46±0.09	0.50±0.06	0.62±0.06‡	0.70±0.09 ‡	1.15±0.21* ‡	1.37±0.20* ‡
Glucose (mmol·l^−1^)	8.64±1.06	7.98±0.71	6.86±0.65	7.49±0.25	11.3±0.70‡	11.41±0.70‡	11.15±0.74‡	11.48±0.70‡
Insulin (ng·ml^−1^)	0.10±0.01	0.19±0.04†	0.12±0.02	0.17±0.03†	0.38±0.04‡	0.40±0.06‡	0.58±0.11* ‡	0.86±0.14* † ‡
HOMA	0.94±0.19	1.63±0.31	0.95±0.22	1.37±0.24	4.56±0.51‡	4.76±0.57‡	7.30±1.74* ‡	10.37±1.57* ‡
Leptin (ng·ml^−1^)	1.34±0.18	1.77±0.25	1.26±0.15	2.03±0.32	9.13±1.24‡	7.54±1.02‡	13.84±2.63* ‡	16.53±1.45* ‡
Adiponectin(ug·ml^−1^)	2.31±0.13	2.83±0.26	2.57±0.23	2.56±0.16	3.79±0.20‡	3.87±0.37‡	4.76±0.33* ‡	5.25±0.33* ‡
Adiponectin(ug·ml^−1^·g^−1^ Rp fat)	0.86±0.09	0.36±0.04†	0.71±0.09*	0.35±0.04†	0.62±0.05‡	0.24±0.03† ‡	0.48±0.03* ‡	0.34±0.04* † ‡
Leptin:adiponectin ratio	0.60±0.08	2.25±0.3†	0.61±0.08	2.14±0.3 †	0.52±0.08 ‡	2.58±0.42† ‡	0.78±0.09* ‡	3.13±0.18* † ‡

Results are expressed as mean±SEM. Data were analysed by multi-factor ANOVA.

P<0.05.

* maternal diet effect, compared with the offspring from chow-fed mothers with the same size litters.

† litter effect, compared with the offspring from mothers fed the same kind of diet with normal size litters.

‡ post-weaning diet effect, compared with chow-fed litter mates.

CN: chow-fed mother with normal size litter.

CS: chow-fed mother with small size litter.

HN: high-fat diet-fed mother with normal size litter.

HS: high-fat diet-fed mother with small size litter.

### Endpoint adiposity and hormone status

At 18 weeks, HFD consumption significantly increased N-A and tibia length, whereas offspring from obese dams had longer tibia independent of diet (P<0.05, Table 3). Liver and heart weights were also greater in rats fed HFD compared with chow-fed litter mates (P<0.05), with over 40% and 50% increase in liver weight in offspring from lean and obese dams, respectively (P<0.001 rats from obese *vs.* lean dams, Table 3). BAT weight was nearly doubled in all HFD fed groups, while white fat mass in all locations sampled was tripled and quadrupled in HFD-fed offspring from lean and obese dams (P<0.05, Table 3), respectively. These differences remained when data were standardized by body weight. Rats from small litters tended to have more adipose tissue and greater liver weight compared with those from normal litters when they consumed chow (Table 3).

Post-weaning HFD feeding interacted with maternal obesity to increase plasma leptin and TG concentrations (P<0.05, HFD effect and interaction with maternal obesity, Table 4); whereas adiponectin was lower and leptin:adiponectin ratio was significantly higher in offspring from small litters (P<0.05, Table 4). Litter size reduction had no impact on plasma leptin and TG levels.

**Figure 4:**
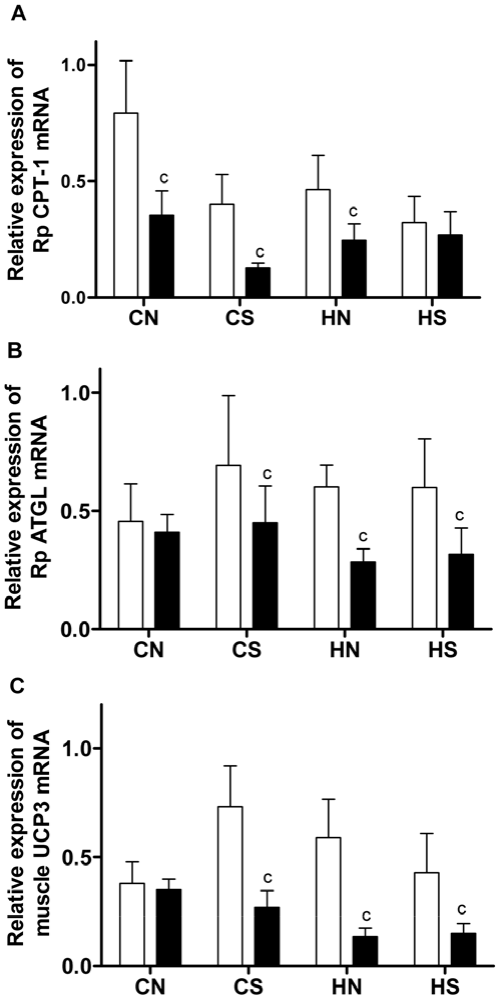
mRNA expression of Rp CPT-1 and ATGL, and muscle UCP3 at 18 weeks. (A) CPT-1 mRNA expression in the Rp fat, (B) ATGL mRNA expression in the Rp fat, (C) UCP3 mRNA expression in the muscle. Results are expressed as mean±SEM (n = 6–8 per group). Data were analysed by multifactor ANOVA. P<0.05, c: post-weaning diet effect, compared with chow-fed litter mates. Post-weaning chow diet: open bars; post-weaning HFD: closed bars. CN: chow-fed mother with normal size litter. CS: chow-fed mother with small size litter. HN: high-fat diet-fed mother with normal size litter. HS: high-fat diet-fed mother with small size litter.

Adipose mRNA expression of CPT-1 and ATGL was reduced in animals fed HFD (Fig. 4A,B); HFD also reduced muscle UCP3 mRNA levels in CSH, HNH and HSH groups (Fig. 4C). At adulthood, neither maternal obesity nor litter size reduction affected markers involved in fatty acid and energy metabolism (CPT-1, ATGL, or UCP3, Fig. 4).

### Hypothalamic markers

**Figure 5:**
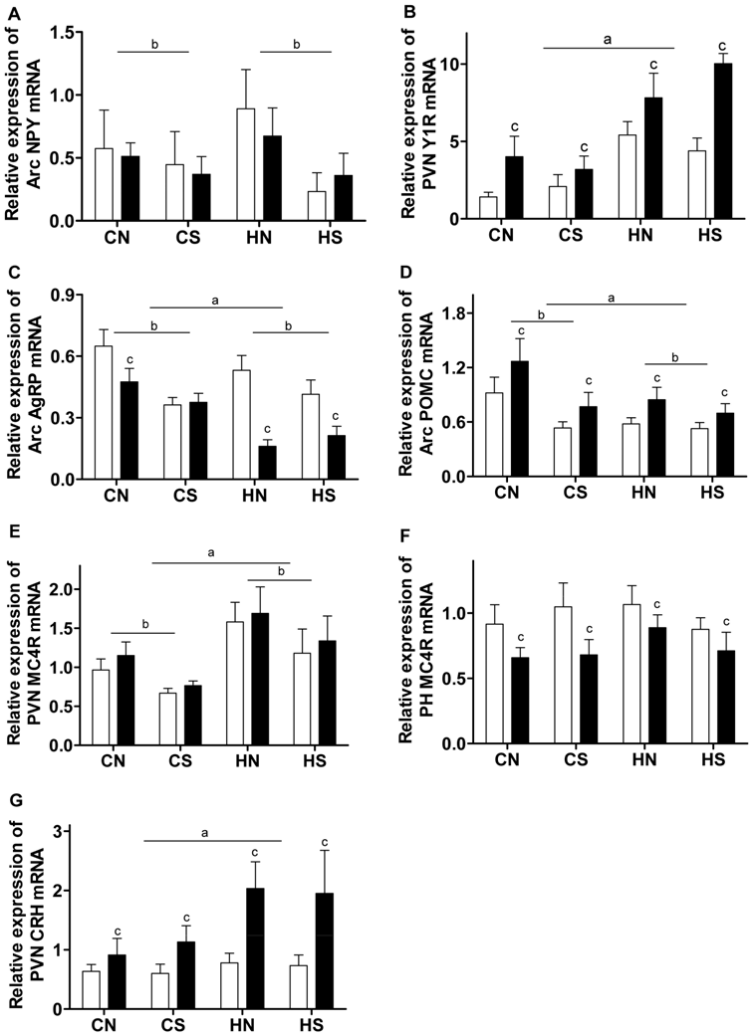
mRNA expression of hypothalamic appetite regulators at 18 weeks. (A) mRNA expression of NPY, (B) Y1 receptor, (C) AgRP, (D) POMC, (E, F) MC4R and (G) CRH in the hypothalamus. Results are expressed as mean±SEM (n = 6–9 per group). Data were analysed by multifactor ANOVA. Post-weaning chow diet: open bars; post-weaning HFD: closed bars. P<0.05, a: maternal diet effect, compared with the offspring from chow-fed mothers with the same size litters; b: litter effect, compared with the offspring from mothers fed the same kind of diet with normal size litters; c: post-weaning diet effect, compared with chow-fed litter mates. CN: chow-fed mother with normal size litter. CS: chow-fed mother with small size litter. HN: high-fat diet-fed mother with normal size litter. HS: high-fat diet-fed mother with small size litter.

Arc NPY mRNA expression was only lower in rats raised in small size litters (P<0.05, litter effect); whereas PVN Y1 receptor mRNA was increased in HFD-fed animals (P<0.05 HFD effects, Fig. 5A,B). Across the 8 groups, there was a positive correlation between energy intake and Y1 receptor mRNA expression (r = 0.76, P<0.05). HFD-feeding interacted with maternal obesity to increase PVN Y1 receptor levels (P<0.05, Fig. 5B). Maternal obesity, litter size reduction and postnatal HFD-consumption all displayed inhibitory effects on Arc AgRP mRNA levels, with more marked effects in HNH and HSH groups (Fig. 5C).

Arc POMC mRNA was increased in all HFD-fed groups (P<0.05, HFD effect); however, it was lower in rats from obese dams or small litters (P<0.05, maternal and litter effects, Fig. 5D). PH MC4R mRNA expression was significantly reduced by HFD consumption, as well as litter size reduction (P<0.05, HFD and litter effects); whereas it was increased by maternal obesity (P<0.05, maternal effect, Fig. 5E,F). PVN CRH was markedly increased in HFD-fed animals, and this was more pronounced in those from obese dams (P<0.05, HFD and interaction with maternal obesity, Fig. 5G).

**Figure 6:**
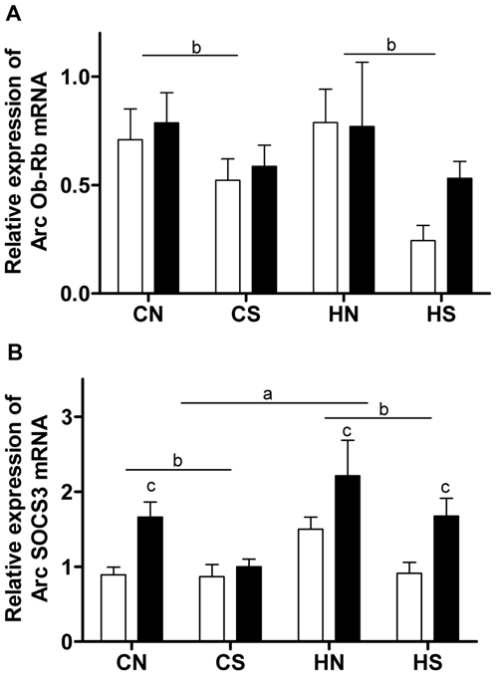
mRNA expression of Ob-Rb and SOCS3 in the Arc at 18 weeks. (A) Ob-Rb mRNA expression, (B) SOCS3 mRNA expression. Results are expressed as mean±SEM (n = 6–8 per group). Data were analysed by multifactor ANOVA. P<0.05, a: maternal diet effect, compared with the offspring from chow-fed mothers with the same size litters; b: litter effect, compared with the offspring from mothers fed the same kind of diet with normal size litters; c: post-weaning diet effect, compared with chow-fed litter mates. Post-weaning chow diet: open bars; post-weaning HFD: close bars. CN: chow-fed mother with normal size litter. CS: chow-fed mother with small size litter. HN: high-fat diet-fed mother with normal size litter. HS: high-fat diet-fed mother with small size litter.

Post-weaning HFD consumption did not have a significant impact on Arc Ob-Rb (Fig. 6) and STAT3 mRNA (not shown), neither did maternal obesity; however, both increased Arc SOCS3 mRNA expression (P<0.05, maternal and HFD effects, Fig. 6). Interestingly, rats from small litters displayed both lower Arc mRNA expression of Ob-Rb and SOCS3 (P<0.05, litter effect, Fig. 6), without significant alteration in STAT3 mRNA (not shown).

## Discussion

The current study investigated the neuroendocrine changes underlying the interaction between post-weaning diets and different levels of pre-weaning overnutrition. Established maternal obesity promoted post-weaning adiposity and glucose intolerance even when offspring consumed low-fat chow. It had a significant additive impact with post-weaning HFD consumption to markedly exaggerate these metabolic disorders in offspring. Although we previously showed immediate postnatal overfeeding due to litter size reduction *per se* had a strong impact on adiposity and interacted with maternal obesity to cause increased adiposity and glucose intolerance during the suckling period [12], here the effect was subsequently overridden by an adverse post-weaning feeding environment. These three interventions impacted differently on hypothalamic and peripheral markers involved in energy homeostasis, suggesting they cause obesity via different mechanisms.

The impact of maternal obesity was passed onto the next generation. Offspring from obese dams had more than 8% higher energy intake than those from lean dams regardless of diet. In humans, if energy intake exceeds energy expenditure by 5%, the resulting annual weight gain of 5 kg [22], if sustained, would lead to frank obesity in just a few years. The small difference (8%) in daily energy intake may explain why offspring from obese dams gained more fat than those from lean dams fed the same diet. As a result, they developed more severe hyperlipidemia, hyperinsulinemia, hyperglycemia, hyperleptinemia, insulin resistance, and glucose intolerance than those from lean dams.

Skeletal muscle has a key role in determining systemic insulin sensitivity, since the majority insulin-stimulated glucose utilization occurs in this tissue [23]. Any reduction of metabolic rate in muscle would reduce glucose utilization, thereby leading to hyperglycemia. In skeletal muscle, adiponectin plays a key role in insulin sensitivity, glucose utilization, and fatty acid oxidation [24], [25], mostly via AdipoR1 [26]. Plasma adiponectin levels were reduced by both maternal obesity and post-weaning HFD consumption when standardized by fat mass, while muscle AdipoR1 was also downregulated by both factors. Adiponectin activity is commonly reduced in human obesity, thus affecting insulin sensitivity [25], [27]. Furthermore, in obese animals, the binding affinity of adiponectin to its receptors was also reduced [27]. Glucose tolerance was reduced in rats consuming HFD, and more markedly in those from obese dams. The plasma leptin:adiponectin ratio is another valid index for insulin resistance [20]. Indeed, in the current study, there was a positive correlation between leptin:adiponectin ratio and body weight, AUC, plasma TG and HOMA, which is consistent with previous studies [20], [21]. This further confirms the close link between adiposity/circulating lipid levels and systemic insulin resistance. Moreover, reduced glucose uptake by cells may be perceived as a fuel deficiency signal by the brain and lead to overeating.

From our observation, rats are born with very little visible white or brown adipose tissues and these develop gradually during the suckling period. This suggests that peripheral markers involved in fatty acid oxidation, especially in fat, are more likely to be regulated by postnatal factors. Indeed, these markers were only significantly inhibited by post-weaning HFD feeding, suggesting that the post-weaning environment is so powerful that it can override any early nutritional programming during early life, as described by others [3], [28]. The universally reduced fatty acid oxidative markers (ATGL, CPT-1 and UCP3) suggest that HFD feeding impaired lipid metabolism to cause fat accumulation. Therefore, rats inevitably lay down extra fat.

NPY expressing neurons are concentrated in the hypothalamic Arc, projecting to PVN to exert their effects [29]. Low hypothalamic NPY levels were consistently observed in our previous studies [6], [7]. The animals in the current study were studied under a fasting state. Our previous study suggests that during fasting, downregulated NPY can be normalized to control levels in the offspring of obese mothers [30]. Therefore, in the fasting state NPY was not different in HFD-fed animals. Activation of the Y1 receptor (major NPY orexigenic receptor [31]) alone can increase food intake [32]. In the current study, the upregulation of PVN Y1 receptor mRNA induced by both maternal obesity and post-weaning HFD consumption could directly contribute to the increased daily energy intake in these animals, as there was a positive correlation between the energy intake and Y1 receptor mRNA expression across groups. These findings are consistent with our earlier report of enhanced responses of NPY signaling to fasting in offspring of obese dams at weaning [30]. Our findings are also in agreement with that of Chang et al, where maternal HFD-feeding from gestational day 6 increased hypothalamic proliferation of different orexigenic peptide-expressing neurons [17].

The adaptive increase in Arc POMC expression in response to post-weaning HFD is consistent with our previous study [33]. It has been suggested that MC4R in different hypothalamic regions serve different functions. PVN MC4R was shown to be involved in feeding regulation, whereas in other hypothalamic regions, MC4R is thought to affect energy expenditure [34]. Thus in the current study, reduced MC4R mRNA in the PH, which contains dorsomedial and ventromedial hypothalamic nuclei, may lead to reduced energy expenditure in HFD-fed rats although energy intake was high. This mismatch caused positive energy balance leading to excessive adipose accumulation [35]. AgRP increases feeding by competing with α-MSH to bind MC4R. AgRP seems to have more plasticity than other appetite regulators measured in this study, since it was reduced by all three over-nutrition states. The downregulation of AgRP may lead to less inhibition of melanocortin signaling to counteract the enhanced NPY signal. The adaptation of POMC and AgRP is possibly an attempt to normalize energy balance. However, fat accumulation and weight gain observed in this study may suggest that the NPY pathway may be more powerful in driving positive energy balance than other central regulators.

There was a reduction in NPY and AgRP expression in animals raised in small litters. This may suggest an adaptation to overcome effects of over-nourishment early in life. However, maternal and early postnatal overnutrition could shift the set point for energy balance and body weight, as observed in adult-onset dietary obesity [36], [37]. Interestingly, Arc POMC and PVN MC4R mRNA expression were simultaneously reduced by litter size reduction, matching the diminished orexigenic effects that would be expected with reduced Arc NPY and AgRP. The universal downregulation of the major appetite regulators is of interest. One explanation could be altered neural density or activity due to excessive nutrition during a critical window of postnatal neural development. NPY and AgRP are key orexigens. NPY binds to its downstream receptors to directly increase feeding; while AgRP increases feeding by competing with α-MSH for binding to MC4R. As such, it inhibits the anorexigenic effect of α-MSH, leading to increased feeding. The downregulation of AgRP may lead to less inhibition of melanocortin signaling, which may have been balanced by the reduced POMC mRNA. However, since Y1 receptor expression was not reduced after litter size reduction, but increased by postnatal HFD, animals still displayed hyperphagia. To prove this hypothesis requires further investigation.

The effects of maternal obesity on body weight gain occurred earlier and lasted longer than litter size reduction, via facilitating activation of orexigenic neural pathways, as evidenced by upregulated PVN Y1 receptor. Indeed, in the current study, offspring from obese dams consumed more when they were fed either chow or HFD. Increased CRH mRNA expression has been observed in palatable HFD fed mice when they were switched to low-fat chow, suggesting increased craving and withdrawal stress [38]. In the current study, maternal obesity and HFD-consumption interacted to increase PVN CRH mRNA expression after overnight food withdrawal. However, further study is needed to follow up our mRNA results at the protein level or against neurotransmitter release.

Leptin is anorexigenic via inhibiting hypothalamic NPY production and its action on Y1 receptor [39]. Leptin resistance is commonly observed in obesity. Overnutrition can blunt central leptin sensitivity before the onset of obesity. Previous studies showed reduced leptin sensitivity in offspring from obese dams [40]. At postnatal day 20, the high plasma leptin in pups from obese dams is suggestive of leptin resistance in our previous study [12], reflected by reduced inhibition of the NPY system upon fasting [30]. In this study, at 18 weeks, maternal impact on plasma leptin levels was still significant in HFD-fed offspring, however not in chow-fed offspring. This suggests that a healthy post-weaning dietary (balanced low-fat chow) intervention can reverse the detrimental maternal impact on adiposity and leptin levels as shown at weaning; whereas consumption of HFD after weaning compounded the maternal impact. Importantly, the combination of maternal obesity and postweaning HFD exerted a greater detrimental impact on metabolism. In the hypothalamus, Ob-Rb was only shown to be reduced by litter size reduction, however increased circulating TG levels in animals consuming HFD may reduce leptin transport across the blood-brain barrier [41]. The increased SOCS3 expression can inhibit leptin signaling, leading to leptin resistance in rats from obese dams and those consuming HFD.

### Conclusion

Overnutrition during different periods of life from the intrauterine and lactation periods through to adulthood caused markedly different changes in the neural markers involved in energy homeostasis. Maternal obesity facilitated brain orexigenic pathways to cause obesity in offspring, while early postnatal over-feeding (litter size reduction) appeared more associated with anorexigenic adaptations, possibly to counteract the over-nutrition, however food intake was not reduced. Moreover our data highlight strong additive detrimental effects of maternal obesity and postweaning HFD consumption to increase the metabolic disorders in offspring. Both nutrition during gestation and postnatal lifestyle should be taken into account when considering optimizing the prevention and management of obesity.
